# In Memoriam: Jay Stephen Keystone (1943–2019)

**DOI:** 10.3201/eid2601.191500

**Published:** 2020-01

**Authors:** David O. Freedman

**Affiliations:** University of Alabama at Birmingham, Birmingham, Alabama, USA

**Keywords:** Jay Keystone, obituary, funny guy, GeoSentinel Surveillance Network, Canada, tropical medicine, travel medicine

After a year-long struggle with cancer, Jay S. Keystone, CM, MD, MSc (TM London) died at 76 years of age on September 3, 2019, surrounded by his devoted wife Margaret and his 5 beloved children (Figure). He talked about his children constantly, even in academic presentations in front of thousands of strangers. Jay always said, “Humor is an important tool in the practice of medicine. In teaching, I use it to engage the learner; in practice, it creates a relationship between me and the patient, which levels the playing field and puts them at ease.” To clinical colleagues, he was the go-to clinician for tropical medicine cases; to trainees, he was the master professor to whom those from all over Canada came to work and learn; and to audiences worldwide, he was a marquee attraction for his legendary humor and wit (*1*). His clever political incorrectness never failed to augment the delivery of the key points on tropical and emerging exotic diseases.

**Figure:**
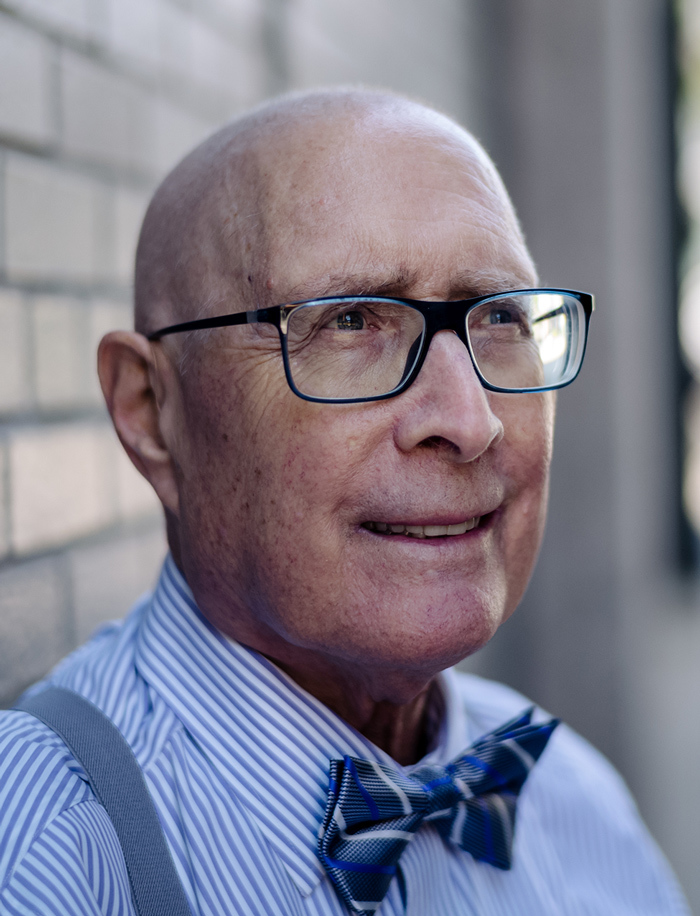
Jay Stephen Keystone (1943–2019)

Jay’s patients worshipped him for his diagnostic acumen and ability to listen. Jay was always in a good mood; full of life; and a caring, warm, and compassionate teacher, mentor, physician, and research collaborator.

As one of Jay’s earliest mentees and professional friends, I appreciated this visionary as a first-year medical student at the University of Toronto in 1977. He was in his first year as director of the Tropical Disease Unit after returning from the London School of Hygiene and Tropical Medicine and fieldwork in Africa and South America. He was clearly on track to lead the divergence of travel medicine from tropical medicine. Air travel was changing that. His famous teaching slide was a response to the question, “What is the most dangerous vector in the world?” The slide answered, “*Anopheles Air canadensi*” and showed the appropriately branded 747 in midflight. He was among the first to recognize that the rapid movement of infected and potentially infectious persons away from the point of infection acquisition would require Keystone-level clinical insights to differentiate the usual tropical diseases from the novel. He imparted to his large cadre of trainees, mentees, and collaborators the use of people skills to forge the links with the laboratory and public health colleagues necessary to complete these puzzles.

Jay authored approximately 200 publications with more than 5,000 citations, remarkable considering the relative obscurity of some of the diseases investigated. His most cited papers were collaborative across multiple disciplines, and none would have been possible without Jay’s acumen at sorting the clinically relevant from the irrelevant. In 1984, working with Canadian surgeon Bernie Langer, Jay published an often-cited case series on the surgical approach to hepatic hydatid disease in immigrants from Greece and Italy to Canada, which forms the basis for our current approaches and understanding (*2*). In 1998, working with Kevin Kain, Jay reported the problems encountered when diagnosing and managing imported malaria, which codified issues we still struggle with today (*3*). A 2004 paper articulated the difficulties of dealing with infection in immigrants and their descendants returning home to visit friends and relatives (*4*). Jay’s legacy includes a superb cadre of clinicians in Toronto, poised in prescient preparation for the next emergence event. Past sentinel events include outbreaks of travelers’ leptospirosis, severe acute respiratory syndrome, Zika, and drug-resistant malaria.

Jay’s vision was instrumental in founding the GeoSentinel Surveillance Network in 1995; while president of the then-fledgling International Society of Travel Medicine, Jay committed a significant portion of the Society’s assets to a $50,000 seed grant to found GeoSentinel. Returning travelers are often sentinels of emerging infectious diseases. GeoSentinel continues to be the largest available database of travel-related illness. With the initial efforts by Phyllis Kozarsky, Hans Lobel, Marty Cetron, and me, and with Jay’s stature behind us, the eventual results were the continuous Centers for Disease Control and Prevention funding of the network to this day. Jay co-authored 2 of the landmark GeoSentinel papers (*5**,**6*).

Of Jay’s many honors, he was most proud of a few. They were the Cody gold medal for standing first in his class through all 4 years of medical school in Toronto, serving as president of the clinical group of the American Society of Tropical Medicine and Hygiene, and receiving the 2008 American Society of Tropical Medicine and Hygiene Ben Kean medal for excellence in teaching and mentoring. In 2015, he was made a Member of the Order of Canada, one of the highest civilian honors in the country, for his outstanding contributions as a pioneer of travel and tropical medicine in Canada.

Jay continued teaching and seeing his patients until a few weeks before his death. He worried about who would follow his cohort of delusional parasitosis patients, a group that few but Jay would listen to and treat with skill and compassion. 

Jay taught me to never take my work overly seriously yet to thrive by enjoying the meaning and relevance of contributions to the health of patients and of the increasingly mobile public. Jay’s life was remarkable not only for his accomplishments and the inspiration that he engendered in others but also for his self-deprecating humor. He maintained close friendships with colleagues from all over the world, who surely share our feelings of loss.
